# Hepatitis B Virus-Specific CD8+ T Cells Maintain Functional Exhaustion after Antigen Reexposure in an Acute Activation Immune Environment

**DOI:** 10.3389/fimmu.2018.00219

**Published:** 2018-02-12

**Authors:** Qin Wang, Wen Pan, Yanan Liu, Jinzhuo Luo, Dan Zhu, Yinping Lu, Xuemei Feng, Xuecheng Yang, Ulf Dittmer, Mengji Lu, Dongliang Yang, Jia Liu

**Affiliations:** ^1^Department of Infectious Diseases, Union Hospital, Tongji Medical College, Huazhong University of Science and Technology, Wuhan, China; ^2^Institute of Infection and Immunology, Union Hospital, Tongji Medical College, Huazhong University of Science and Technology, Wuhan, China; ^3^Institute for Virology, University Hospital of Essen, University of Duisburg-Essen, Essen, Germany

**Keywords:** hepatitis B virus, CD8+ T cell, functional exhaustion, cell transfer, immune environment

## Abstract

Chronic hepatitis B virus (HBV) infection is characterized by the presence of functionally exhausted HBV-specific CD8+ T cells. To characterize the possible residual effector ability of these cells, we reexposed CD8+ T cells from chronically HBV replicating mice to HBV antigens in an acute activation immune environment. We found that after transfer into naive mice, exhausted CD8+ T cells reexpanded in a comparable magnitude as naive CD8+ T cells in response to acute HBV infection; however, their proliferation intensity was significantly lower than that of CD8+ T cells from acute-resolving HBV replicating mice (AR mice). The differentiation phenotypes driven by acute HBV replication of donor exhausted and naive CD8+ T cells were similar, but were different from those of their counterparts from AR mice. Nevertheless, exhausted CD8+ T cells maintained less activated phenotype, an absence of effector cytokine production and poor antiviral function after HBV reexposure in an acute activation immune environment. We thus conclude that exhausted CD8+ T cells undergo a stable form of dysfunctional differentiation during chronic HBV replication and switching immune environment alone is not sufficient for the antiviral functional reconstitution of these cells.

## Introduction

Hepatitis B virus (HBV) infection continues to be a major cause of chronic liver diseases worldwide (1). The outcomes of HBV infection vary greatly from person to person. Some subjects control infection efficiently and clear the virus either with or without a clinically evident liver inflammation (2). Other patients fail to clear the virus and develop chronic infection, which often lead to the development of cirrhosis and hepatocellular carcinoma (3, 4).

There is consensus that adaptive immune responses, especially that of virus-specific CD8+ T cells, play major roles in the defense against HBV infection (5). For instance, a vigorous polyclonal and multispecific CD8+ T cell response was detectable in the peripheral blood of patients with acute hepatitis B (6). Experimental depletion of CD8+ T cells significantly delayed HBV clearance in infected chimpanzees (7). In contrast, chronic HBV infection is characterized by weak or undetectable HBV-specific CD8+ T cell responses and the presence of functionally exhausted HBV-specific CD8+ T cells that are unable to clear the virus. It has been demonstrated that multiple mechanisms may contribute to the dysfunction of HBV-specific T cells in chronic HBV infection, such as persistent high viral load and high antigen levels, sustained expression of multiple inhibitory molecules including PD-1, TIM-3, LAG-3, CTLA-4, and 2B4, and excessive immunosuppressive signals encountered in the liver microenvironment (8–11).

Recently, accumulating studies have suggested that although virus-specific CD8+ T cells become exhausted and exhibit poor effector functions, they can still control viral replication during chronic viral infection (12, 13). For instance, the elimination of CD8+ T-cells from Simian immunodeficiency virus-infected Rhesus macaques resulted in a prominent increase in viral load (14). In mice that were chronically infected with lymphocytic choriomeningitis virus (LCMV), a subset of exhausted CD8+ T cells expressing the chemokine receptor CXCR5 has been found playing a critical role in the control of viral replication (12). Moreover, the recovery of T cell functions after blockade of inhibitory receptors such as PD-1, CTLA-4, 2B4, and TIM-3 indicates that T cell responses are downregulated rather than being terminated during chronic infection (11, 15–17). The suppression of HBV-specific T cell responses is more profound in patients with higher viral titers, indicating ongoing immune control by the virus-specific T cells in chronic HBV infection (13, 18). However, more direct investigation is absent either to support the claim or to reveal the effector state of exhausted HBV-specific CD8+ T cells in detail.

In this study, we characterized the possible residual effector ability of functionally exhausted HBV-specific CD8+ T cells by using the HBV hydrodynamic injection (HI) mouse model. The CD8+ T cells from chronically HBV replicating, acute-resolving HBV replicating, and naive mice were reexposed to HBV antigens in an acute activation immune environment and were examined for their proliferation, activation, differentiation, and antiviral function, which allowed us to investigate the effector state of exhausted HBV-specific CD8+ T cells in detail.

## Materials and Methods

### Mice

Male, 6- to 8-week-old, CD45.2 wild-type C57BL/6 mice were purchased from Hunan Slack King Laboratory Animal Co., Ltd. (Changsha, China). CD45.1 congenic mice were purchased from the Jackson Laboratory. All animals were maintained under specific pathogen-free (SPF) conditions in the Animal Care Center of Tongji Medical College.

### HBV Replication Mouse Model

Hydrodynamic tail vein injection is a simple, safe, and effective method for gene transfer into small rodents and has been successfully applied to deliver HBV genome into murine hepatocytes, resulting in production of viral antigens, replicative intermediates, and viral particles (19, 20). The HBV HI mouse model affords the opportunity to identify and characterize the immunological events related to HBV infection (21). HI was performed as described previously by using HBV plasmids pSM2 (provided by Dr. Hans Will, Heinrich-Pette-Institute, Hamburg, Germany) and pAAV/HBV1.2 (provided by Professor Pei-Jer Chen, National Taiwan University College of Medicine, Taipei, Taiwan) to establish HBV replication in mice (22–24). In brief, male mice at 6 to 8 weeks of age were injected with 10 µg pSM2 or pAAV/HBV1.2 in a volume of normal saline solution equivalent to 0.1 ml/g of the mouse body weight through the tail vein within 8 s.

### Isolation of Lymphocytes from the Spleen and Liver

Preparation of single-cell suspensions of murine splenocytes was performed according to a protocol described previously (25). Mouse intrahepatic lymphocytes (IHLs) were isolated as described previously (23). In brief, the mouse liver was perfused *via* the portal vein with 10 ml PBS immediately after sacrificing. After perfusion, the liver was homogenized and digested with enzyme solution containing 0.05% collagenase type IV (Sigma-Aldrich), 0.002% DNAase I (Sigma-Aldrich), and 10% fetal bovine serum for 30 min. The pellet after digestion was resuspended in 40% Percoll and centrifuged at 1,000 *g* for 15 min without braking. After removing the debris and hepatocytes on the top layer, IHLs in the pellet were collected, washed, and subjected to further analysis.

### Adoptive CD8+ T Cell Transfer

CD8+ T cell isolation was performed by magnetic activated cell sorting using a mouse CD8a+ T cell isolation kit (Miltenyi Biotec). The purity of CD8+ T cells was above 90% after isolation (Figure S1 in Supplementary Material). 5 × 10^6^ splenic CD8+ T cells from naive, pAAV/HBV1.2-injected or pSM2-injected CD45.2 mice were suspended, respectively, in 500 µl sterile PBS and were injected into naive CD45.1 recipient mice through the tail vein.

### Detection of Serological HBV Antigen and HBV DNA

Sera were prepared from blood collected from the retro-orbital sinus of the mouse at the indicated time points. Serum levels of HBsAg and HBeAg were measured by the corresponding ELISA kits (Kehua, Shanghai, China), according to the manufacturer’s instructions. HBV DNA copies were measured by a diagnostic kit for HBV DNA (Sansure, Changsha, China) using quantitative real-time polymerase chain reaction according to the manufacturer’s instructions.

### Detection of HBV-Specific CD8+ T Cells by Dimer Staining

Hepatitis B virus-specific CD8+ T cells were detected using soluble DimerX H-2Kb:Ig fusion protein technology (BD Biosciences) according to the manufacturer’s instructions. Briefly, 0.8 µg dimer per sample was loaded with 2.4 μg H-2Kb-restricted HBcAg-derived CD8+ epitope peptide core93-100 (MGLKFRQL) at 4°C for 24 h. Freshly isolated lymphocytes were firstly incubated with purified anti-mouse CD16/32 antibody (Biolegend) to block their FcRs at 4°C for 10 min, and then were incubated with peptide-loaded or unloaded dimer at 4°C for 1 h. The peptide-unloaded dimer staining served as a negative control. PE- or FITC-conjugated anti-mouse IgG1 antibody was used to label the H-2Kb:Ig dimer molecule by incubating at 4°C for 20 min. The background levels of the dimer staining in the splenocytes of naive mice were about 0.2% for FITC labeled dimer and were about 0.4% for PE labeled dimer (Figure S2A in Supplementary Material).

### *In Vitro* Stimulation of Murine Lymphocytes

Freshly isolated liver infiltrated lymphocytes or splenocytes were stimulated with 10 μg/ml H-2Kb-restricted HBcAg-derived CD8+ epitope peptide core93-100 (MGLKFRQL), or HBsAg-derived CD8+ epitope peptide env208-216 (ILSPFLPLL) at 37°C for 5 h, in the presence of 1 µg/ml anti-CD28 antibody (eBioscience) and 3 µg/ml Brefeldin A (eBioscience). Cells without peptide stimulation served as a negative control. Cells stimulated with 50 ng/ml PMA and 1 µg/ml ionomycin (Sigma-Aldrich) served as a positive control. The background levels of the assay for all three cytokines were less than 0.2% (Figure S2B in Supplementary Material).

### Flow Cytometry

Surface and intracellular staining for flow cytometry analysis were performed as described previously (23, 26). The antibodies used for surface staining included APC-Cy7-anti-CD4, Pacific Blue-anti-CD8a, APC-anti-CD44, APC-anti-PD-1, PerCP-Cy5.5-anti-CD43, PE-Cy7-anti-CD62L, PE-anti-CTLA-4, PE-anti-LAG-3 (all from BD Biosciences), PE-anti-CD45.1, PE-anti-CD45.2, and PE-Cy7-anti-CD45.2 (all from BioLegend). For intracellular cytokine staining, cells were fixed and permeabilized using the Intracellular Fixation and Permeabilization Buffer Set (Invitrogen) and were stained with APC-anti-interferon (IFN)γ, PerCP-Cy5.5-anti-interleukin (IL)-2, FITC-anti-tumor necrosis factor (TNFα) (from BD Bioscience). For Ki67 staining, cells were fixed and permeabilized using the True-Nuclear Transcription Factor Buffer Set (Biolegend) and were stained with BV421-anti-Ki-67 (Biolegend). Freshly isolated cells were used for all assays and about 20,000–40,000 T cells were acquired for each sample using BD FACSCanto II flow cytometer. Data analysis was performed using FlowJo software (Tree Star, Ashland, OR, USA). Cell debris and dead cells were excluded from the analysis based on scatter signals and Fixable Viability Dye eFluor 506 (eBioscience).

### Statistical Analysis

Statistical analyses were performed using the SPSS statistical software package (version 22.0, SPSS Inc., Chicago, IL, USA). The Shapiro–Wilk method was used for normality test. Parametric analysis methods were used when the data were normally distributed; otherwise, nonparametric tests were employed. Statistical differences were analyzed by parametric one-way analysis of variance (ANOVA) test with Least Significant Difference (LSD) *post hoc* test for multiple comparisons and nonparametric Kruskal–Wallis test with Dunn’s *post hoc* multiple comparisons test, when appropriate. Repeated measures ANOVA followed by Tukey’s multiple comparisons test was used to evaluate the statistical differences of the viral loads changes in mice transferred with different cells. Meanwhile, analysis of covariance (ANCOVA) was employed to compare the viral load at the same time point with adjusting for the confounding effects of the former time among the different groups. All reported *P* values were two-sided, and a *P*-value less than 0.05 was considered statistically significant.

### Ethics Statement

All animal experiments were conducted in a BSL-2 laboratory facility in accordance with the Guide for the Care and Use of Laboratory Animals and were reviewed and approved by the Institutional Animal Care and Use Committee at Tongji Medical College, Huazhong University of Science and Technology, China (IACUC Number: 612).

## Results

### Exhausted CD8+ T Cells Maintain Poor Anti-HBV Function after HBV Reexposure in an Acute Activation Immune Environment

**Figure 1:**
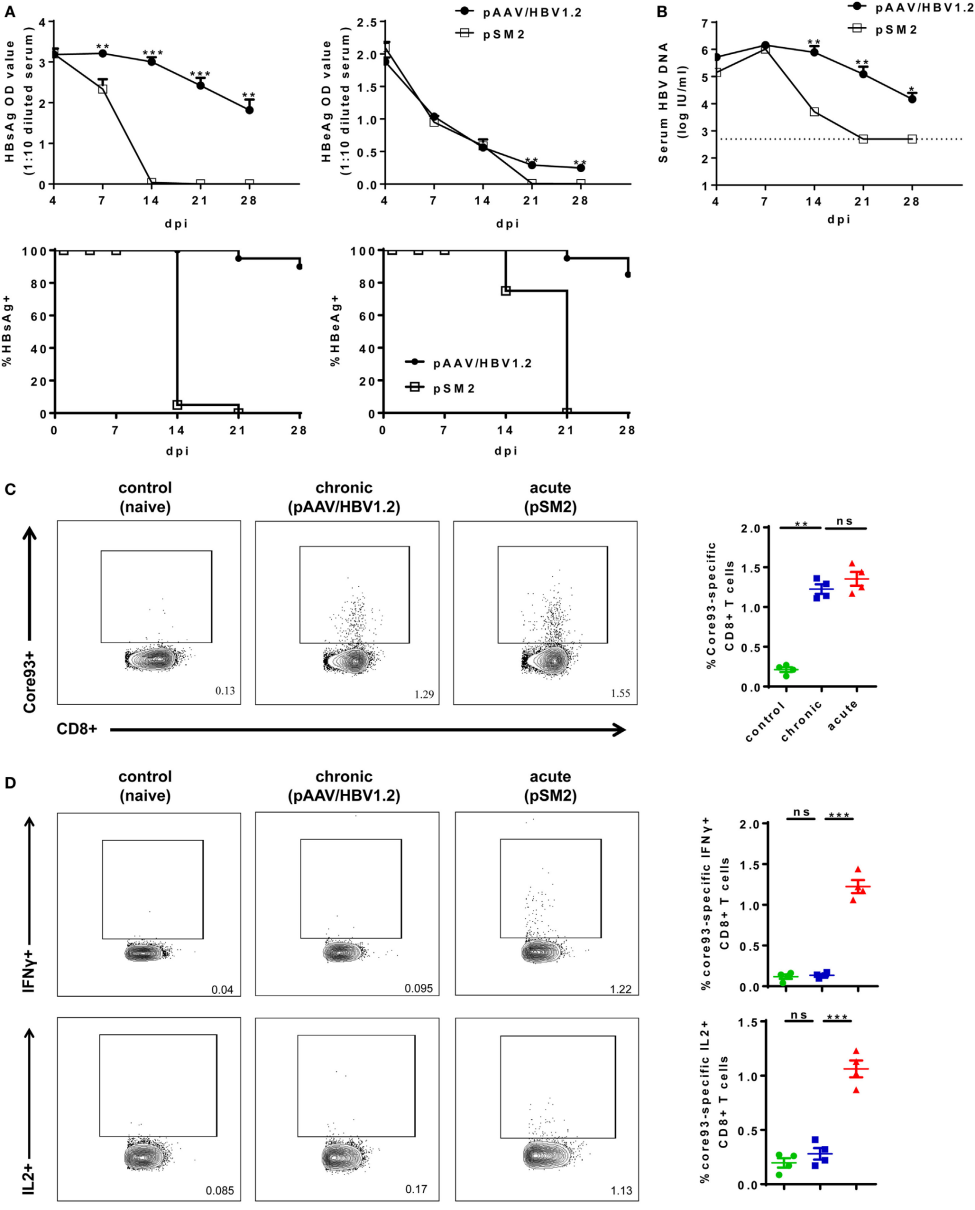

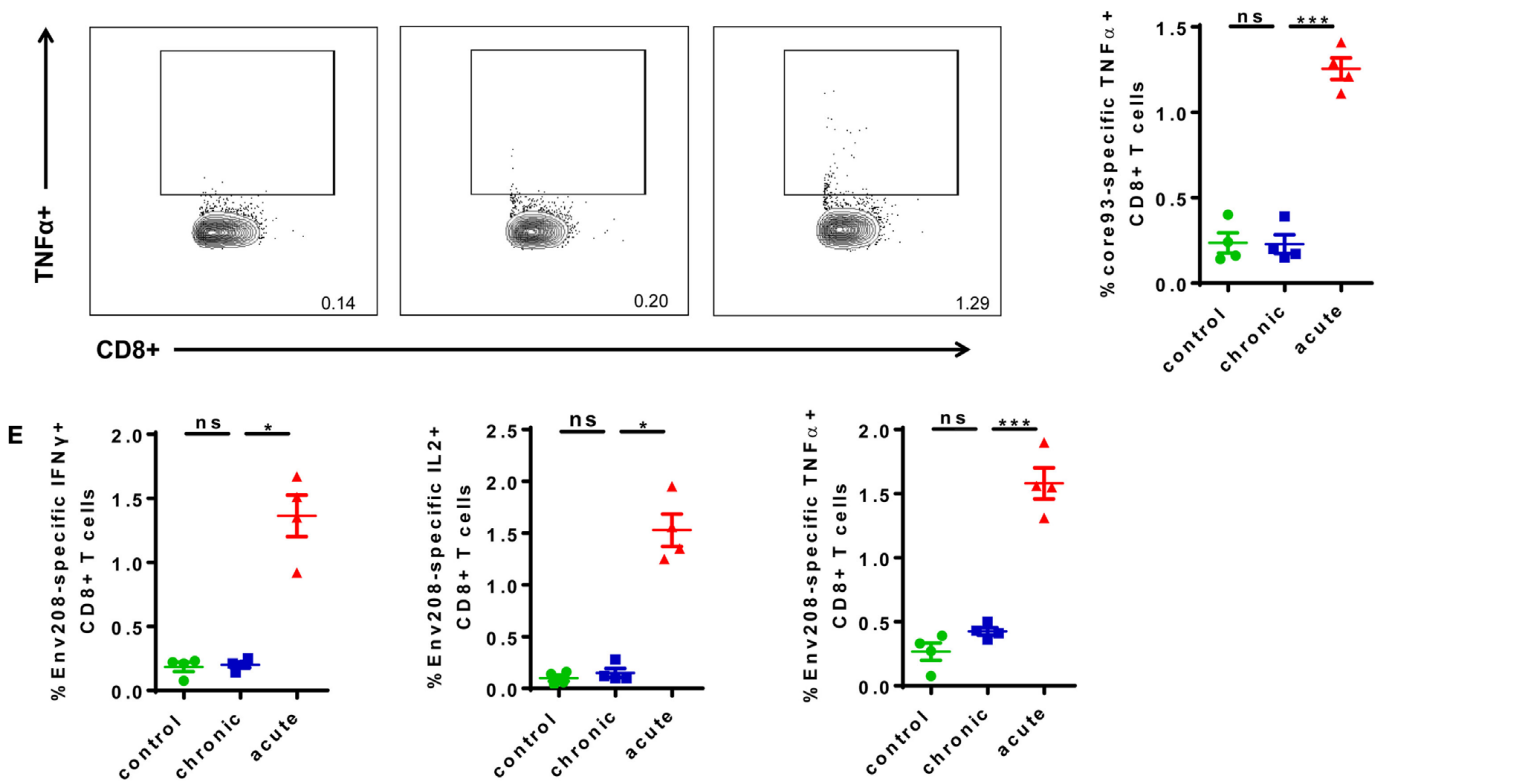
Characterization of hepatitis B virus (HBV)-specific CD8+ T cells in the spleen of chronically HBV replicating mice and acute-resolving HBV replicating mice. C57BL/6 mice were hydrodynamically injected with either pAAV/HBV1.2 or pSM2 plasmid. **(A)** The levels of serum HBsAg and HBeAg in the plasmid injected mice were monitored at indicated time points. The data were analyzed by analysis of covariance. **(B)** The serum HBV DNA levels in mice were monitored at indicated time points. The data were analyzed by repeated measures analysis (*P* < 0.05). **(C)** Frequencies of HBV core93-specific CD8+ T cells were detected in the spleen of plasmid injected mice at day 21 after hydrodynamic injection by FITC dimer staining. **(D,E)** Splenocytes were separated from plasmid injected mice at day 21 and were stimulated with core93 or env208 peptide for 5 h *in vitro*. Intracellular staining was performed and the frequencies of interferon-γ+, interleukin-2+, and tumor necrosis factor-α+ CD8 T cells are shown. Splenocytes from naive mice were used as a negative control. Data are representative of three independent experiments. One-way ANOVA followed by least significant difference test were applied. *P < 0.05; **P < 0.01; ***P < 0.001; ns, not significant.

To experimentally mimic human HBV infection, we utilized HBV HI mouse model. We performed HI with plasmids pSM2 and pAAV/HBV1.2 in male C57BL/6 mice and monitored serum HBsAg, HBeAg, and HBV DNA levels for 28 days postinjection (dpi). pSM2 is a pUC19 vector-based plasmid harboring a head-to-tail tandem dimeric HBV genome (27), which has been shown by us and other groups previously to cause acute-resolving HBV replication in mice after HI (22, 23, 28–30). pAAV/HBV1.2 is a pAAV-GFP vector-based plasmid harboring 1.2-fold of HBV genome (spanning nucleotides 1,400 ~ 3,182/1 ~ 1,987) (22). HI of pAAV/HBV1.2 causes chronic HBV replication in mice (22, 30). The plasmid backbone has been shown to play an important role in determining HBV persistence in pAAV/HBV1.2 HI mice, as the replacement of pAAV vector by another vector resulted in only transient HBV antigenemia. Nevertheless, the cellular immunity against HBV is the key factor, which determines HBV clearance in the HI mouse model (21, 22). As shown in Figure 1, all mice injected with pSM2 plasmid were negative for serum HBsAg, HBeAg, and HBV DNA at 21 dpi. In contrast, pAAV/HBV1.2 plasmid injected mice remained positive for serum HBsAg, HBeAg, and HBV DNA at 21 dpi (Figures 1A,B). Next, we analyzed the phenotypes of splenic CD8+ T cells in acute-resolving HBV-replicating (AR) and chronically HBV-replicating (CH) mice at 21 dpi. No significant differences in the expression of inhibitory receptors, such as PD-1, CTLA-4, and LAG-3 on CD8+ T cells were observed among naive, AR, and chronically HBV replicating mice (CH mice) (Figure S3A in Supplementary Material). However, CH mice showed significantly lower CD43 expression on CD8+ T cells compared to acute-resolving HBV replicating mice (AR mice), indicating these cells maintained a less activated status (Figure S3A in Supplementary Material). Moreover, both CH and AR mice showed decreased naive CD8+ T cell frequencies and increased effector memory T cell (T_EM_) frequencies compared to naive mice. However, the frequencies of central memory T cells (T_CM_) in CH mice were significantly lower than those in AR mice and were at a comparable level to those in naive mice (Figure S3B in Supplementary Material). The frequencies of CD44− CD62L− T cells in CH mice were significantly higher than those in AR and naive mice (Figure S3B in Supplementary Material). Next, we analyzed the frequencies and functions of HBV-specific CD8+ T cells in AR and CH mice. Comparable levels of core93-specific CD8+ T cells were detected in the spleens of AR and CH mice (Figure 1C). However, in contrast to the observations in AR mice, the core93-specific CD8+ T cells from CH mice showed little production of IFN-γ, IL-2, and TNF-α in response to the epitope peptide stimulation (Figure 1D). Similar results were observed for the env208-specific CD8+ T cell response in AR and CH mice (Figure 1E). These results support the conclusion of previous reports that HBV-specific CD8+ T cells are functionally exhausted during chronic HBV infection (15, 18, 31–33).

**Figure 2:**
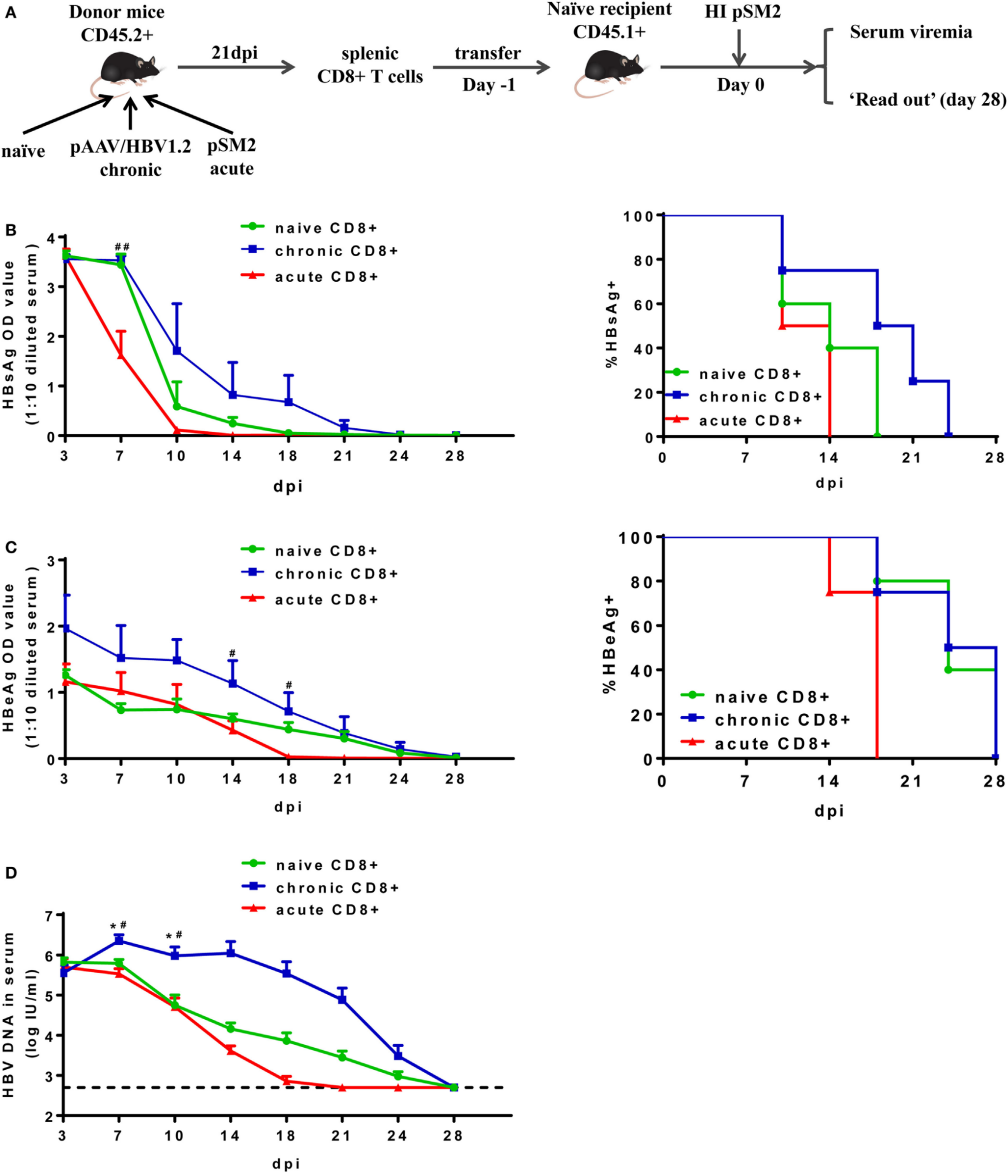
Analysis of antiviral function of CD8+ T cells from chronically hepatitis B virus (HBV) replicating mice. **(A)** Scheme of cell transfer and HBV hydrodynamic injection. Splenic CD8+ T cells from naive, chronically or acutely resolving HBV replicating mice were adoptively transferred into naive mice. Recipients were hydrodynamically injected with pSM2 1 day post cell transfer and were monitored for serum viremia. The kinetics of serum HBsAg **(B)**, HBeAg **(C)**, and HBV DNA levels **(D)** are shown and the statistical differences were analyzed by repeated measures analysis. The asterisks mark the statistically significant differences between CT and NT mice. The hash signs mark the significant differences between CT and AT mice. Data are representative of two independent experiments. *P < 0.05; ^#^*P* < 0.05; ^##^*P* < 0.01.

Next, we isolated total CD8+ T cells from the spleens of CD45.2+ mice at 21 dpi after either pAAV/HBV1.2 or pSM2 plasmid HI, and transferred these cells into CD45.1+ congenic naive mice. Transferring splenic CD8+ T cells from naive CD45.2+ mice served as a control. The recipient mice were then hydrodynamically injected with pSM2 plasmid and monitored for viremia as well as CD8+ T cell responses (Figure 2A). The clearances of serum HBsAg, HBeAg, and HBV DNA were significantly delayed in mice transferred with CD8+ T cells from CH mice (CT mice) compared to those in mice transferred with CD8+ T cells from AR mice (AT mice) (Figure 2B, left panel, HBsAg, *P* = 0.017; Figure 2C, left panel, HBeAg, *P* = 0.049; Figure 2D, HBV DNA, *P* = 0.021). The clearance of serum HBV DNA, but not HBsAg and HBeAg, was less efficient in CT mice compared to that in mice transferred with naive CD8+ T cells (NT mice) (Figure 2B, left panel, HBsAg, *P* = 0.172; Figure 2C, left panel, HBeAg, *P* = 0.078; Figure 2D, HBV DNA, *P* = 0.033). CT mice showed significantly higher HBsAg levels at 7 dpi and HBeAg levels at 14 and 18 dpi than AT mice (Figures 2B,C, left panel). CT mice also showed significantly higher HBV DNA levels at 7 and 10 dpi than both NT and AT mice (Figure 2D). All AT mice became serum HBsAg negative at 14 dpi and HBeAg negative at 18 dpi, while 75% of CT mice remained HBsAg-positive and HBeAg-positive at corresponding time points (Figures 2B,C, right panel). This result indicates that the CD8+ T cells from CH mice maintain poor antiviral function *in vivo* after reexposure to antigens in acute HBV infection setting.

### Reexpansion of Exhausted CD8+ T Cells after HBV Reexposure in an Acute Activation Immune Environment

**Figure 3:**
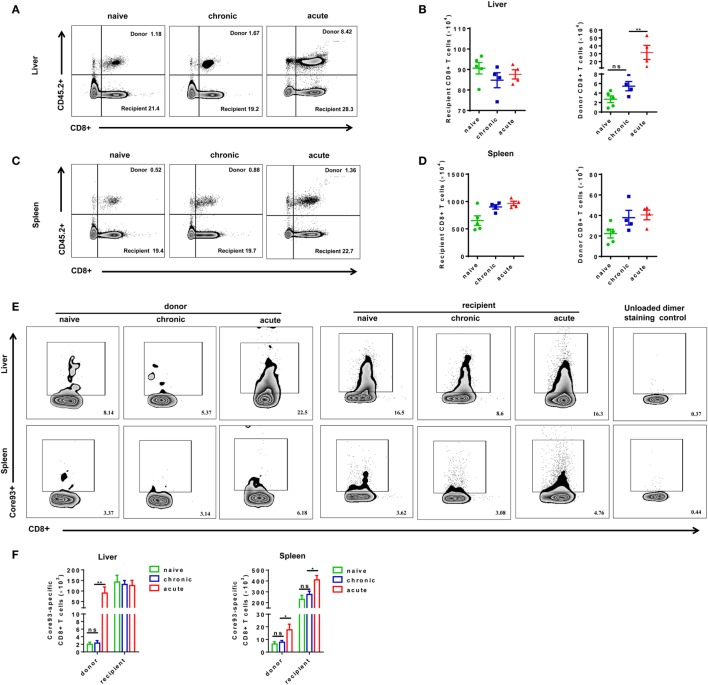
Analysis of CD8+ T cell absolute numbers after cell transfer and hepatitis B virus hydrodynamic injection. Splenic CD8+ T cells from chronically HBV replicating mice, acute-resolving HBV replicating mice, or naive mice (CD45.2+) were adoptively transferred into naive mice (CD45.1+). Recipients were hydrodynamically injected with pSM2 and were sacrificed at day 28. Representative dot plots show the percentages of donor and recipient CD8+ T cells in the liver **(A)** and spleen **(C)**. The absolute numbers of recipient and donor CD8+ T cells in the liver **(B)** and spleen **(D)** were analyzed by flow cytometry. **(E)** Representative dot plots show the frequencies of core93-specific CD8+ T cells in the liver and spleen. **(F)** The absolute numbers of donor and recipient core93-specific CD8+ T cells in the liver and spleen were analyzed by PE dimer staining method. **(A,B)** Representative results from three independent experiments are shown. Kruskal–Wallis test followed by Dunn’s *post hoc* tests were applied. **(C–F)** Representative results from three independent experiments are shown. One-way ANOVA with least significant difference *post hoc* tests were used. *P < 0.05; **P < 0.01; ns, not significant.

Next, we examined whether exhausted CD8+ T cells from CH mice possess the capacity of expansion after reexposure to HBV in acute HBV infection setting. The absolute numbers of total and HBV-specific CD8+ T cells in the liver and the spleen of mice were analyzed at 28 dpi after secondary HBV exposure. All mice showed no significant difference in the absolute numbers of recipient CD8+ T cells in the liver (Figures 3A,B). The number of donor CD8+ T cells in the liver of CT mice was about twofold to that of NT mice; however, the difference was not statistically significant (Figures 3A,B). AT mice showed a profound increase in donor cell numbers in the liver compared to NT and CT mice (Figure 3B). In contrast to the observations in the liver, we did not observe significant differences in numbers of CD8+ T cells in the spleen among NT, CT, and AT mice (Figures 3C,D). The numbers of donor core93-specific CD8+ T cells of AT mice were significantly higher than those of NT and CT mice in both the spleen and liver. No significant differences in numbers of donor core93-specific CD8+ T cells in the liver and spleen were observed between NT and CT mice (Figures 3E,F).

**Figure 4:**
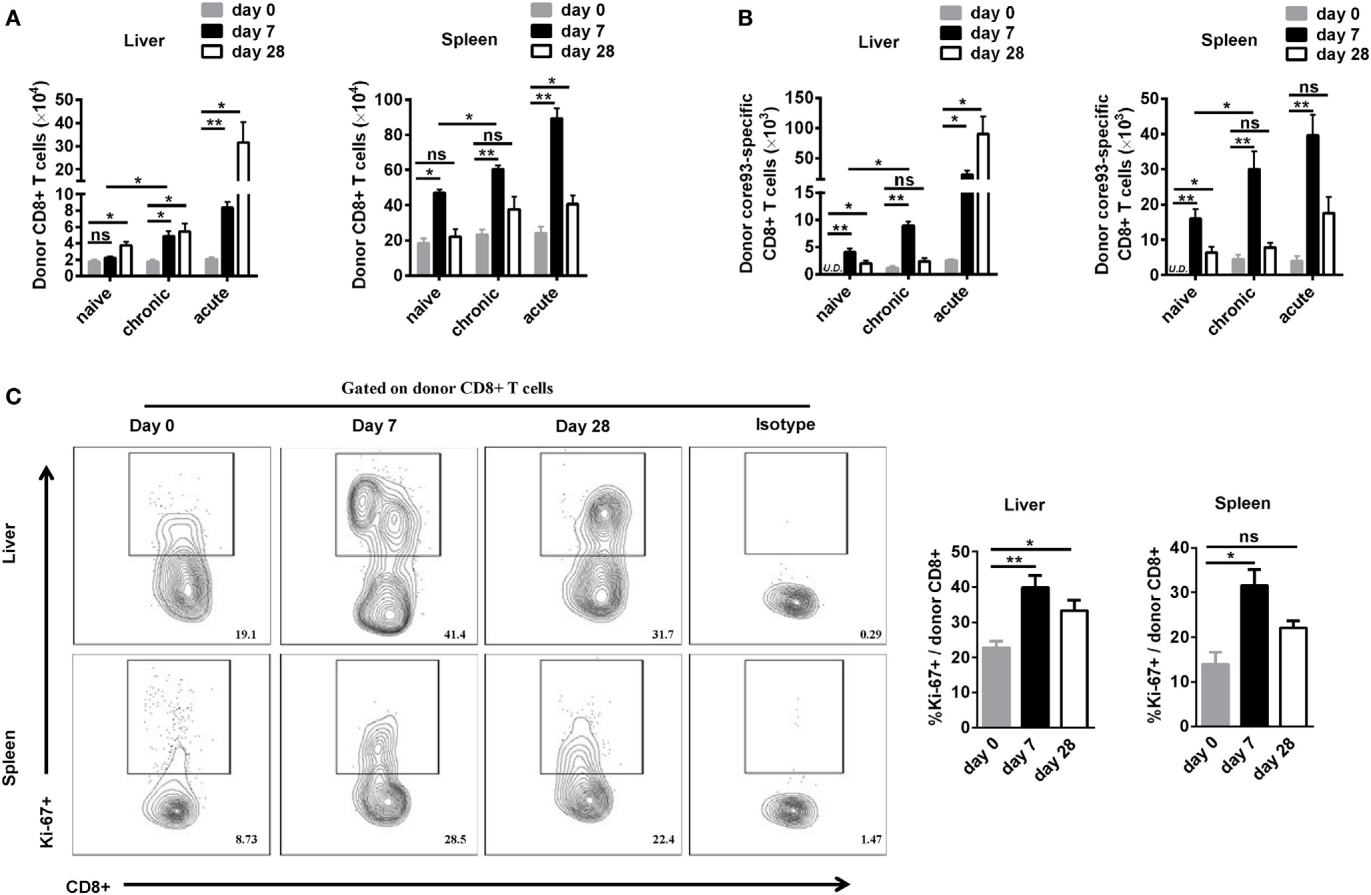
Analysis of the proliferation of engrafted CD8+ T cells after cell transfer and hepatitis B virus hydrodynamic injection. Splenic CD8+ T cells from chronically HBV replicating mice, acute-resolving HBV replicating mice, or naive mice (CD45.2+) were adoptively transferred into naive mice (CD45.1+). Recipients were hydrodynamically injected with pSM2. The kinetic changes of absolute numbers of engrafted CD8+ T cells **(A)** and core93-specific CD8+ T cells **(B)** in the liver and the spleen were analyzed by flow cytometry at the indicated time points. **(C)** Kinetics of Ki-67 expression in donor CD8+ T cells in the liver and the spleen of CT mice were analyzed by flow cytometry. Data are representative of two independent experiments. One-way ANOVA with least significant difference *post hoc* tests were applied. *P < 0.05; **P < 0.01; ns, not significant.

To further characterize the proliferation status of transferred cells, we analyzed the kinetic changes of the absolute numbers of engrafted CD8+ T cells as well as core93-specific CD8+ T cells in the liver and spleen after transfer (day 0) and 7 days after secondary HBV HI (day 7). The “take” rates of engrafted cells by the hosts were about 0.35% in the liver and about 4% in the spleen at day 0. No significant differences of the “take” rates were observed among NT, CT, and AT mice (Figure S4 in Supplementary Material). The absolute numbers of both engrafted total CD8+ T cells and core93-specific CD8+ T cells significantly increased at day 7 than at day 0 in the liver and spleen in CT mice (Figures 4A,B). Importantly, CT mice showed significantly increased numbers of engrafted total CD8+ T cells and core93-specific CD8+ T cells than NT mice at day 7 in both the liver and the spleen (Figures 4A,B), indicating the expansion of engrafted memory T cells rather than naive T cells in CT mice. Moreover, the proliferation of engrafted CD8+ T cells in CT mice was examined by Ki67 staining. A significant increase in Ki67+ expression of engrafted CD8+ T cells was observed in CT mice in both the liver and spleen at day 7 compared to that at day 0 (Figure 4C).

Taken together, these observations indicated that the exhausted CD8+ T cells from CH mice reexpanded in response to HBV reexposure in an acute activation immune environment, but the proliferation intensity was significantly lower than that of their counterparts from AR mice.

### Phenotypes of Exhausted CD8+ T Cells from CH Mice after HBV Reexposure in an Acute Activation Immune Environment

**Figure 5:**
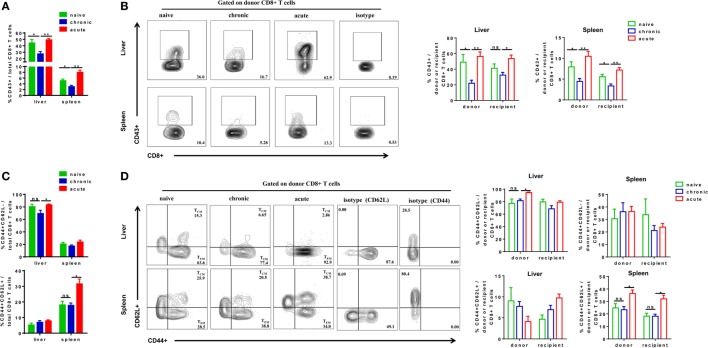
Analysis of CD8+ T cells phenotype after cell transfer and hepatitis B virus hydrodynamic injection. Splenic CD8+ T cells from chronically HBV replicating mice, acute-resolving HBV replicating mice, or naive mice (CD45.2+) were adoptively transferred into naive mice (CD45.1+). Recipients were hydrodynamically injected with pSM2 and were sacrificed at day 28. The frequencies of CD43+ total **(A)**, donor, and recipient **(B)** CD8+ T cells in the liver and the spleen were analyzed by flow cytometry. The frequencies of CD44+CD62L− T_EM_ cells and CD44+CD62L+ T_CM_ cells of total **(C)**, donor, and recipient **(D)** CD8+ T in the liver and the spleen were analyzed by flow cytometry. Data are representative of three independent experiments. Statistics analysis was performed by one-way ANOVA with least significant difference *post hoc* tests. *P < 0.05; **P < 0.01; ns, not significant.

We next examined the phenotypes of activation and differentiation of donor-derived and endogenous CD8+ T cells after cell transfer and HBV challenge. Total CD8+ T cells in CT mice showed significantly lower expression of activation marker CD43 compared with those in NT and AT mice (Figure 5A). The CD43 expressions of donor CD8+ T cells of CT mice were also significantly lower than those of NT and AT mice in both the liver and the spleen (Figure 5B). Interestingly, we observed that transferring CD8+ T cells from CH mice resulted in a significant decrease of CD43 expression on recipient CD8+ T cells in the spleen compared to those of NT and AT mice (Figure 5B). We also examined the differentiation of CD8+ T cells by analyzing CD44 and CD62L expression on the cells. T_EM_ were identified as CD44+CD62L−, while T_CM_ were CD44+CD62L+ (34–37). The major population of CD8+ T cells found in the liver of all groups was with T_EM_ phenotype (Figure 5C). No significant differences in T_EM_ and T_CM_ frequencies of total, donor, and recipient CD8+ T cells were found in the liver and the spleen between CT and NT mice (Figures 5C,D). However, CT mice showed significantly lower T_EM_ frequencies of total and donor CD8+ T cells in the liver and significantly lower T_CM_ frequencies of total, donor, and recipient CD8+ T cells in the spleen than AT mice (Figures 5C,D).

**Figure 6:**
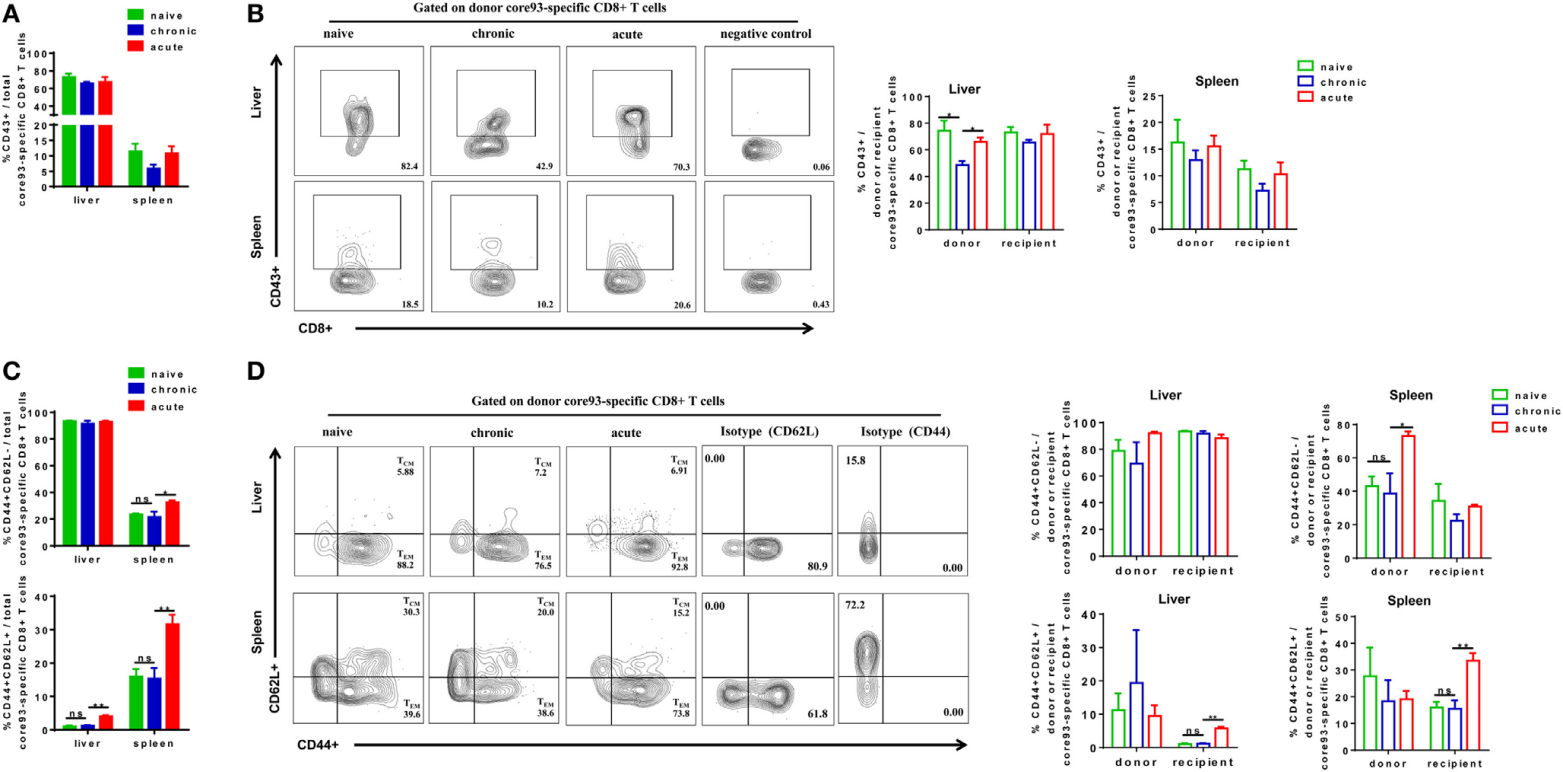
Analysis of the phenotype of core93-specific CD8+ T cells after cell transfer and hepatitis B virus hydrodynamic injection. Splenic CD8+ T cells from chronically HBV replicating mice, acute-resolving HBV replicating mice, or naive mice (CD45.2+) were adoptively transferred into naive mice (CD45.1+). Recipients were hydrodynamically injected with pSM2 and were sacrificed at day 28. The frequencies of CD43+ total **(A)**, donor, and recipient **(B)** core93-specific CD8+ T cells examined by PE dimer staining in the liver and spleen were analyzed by flow cytometry. The frequencies of CD44+CD62L− T_EM_ cells and CD44+CD62L+T_CM_ cells of total **(C)**, donor, and recipient **(D)** core93-specific CD8+ T examined by PE dimer staining in the liver and spleen were analyzed by flow cytometry. Data are representative of three independent experiments. Statistics analysis was performed by one-way ANOVA with least significant difference *post hoc* tests. *P < 0.05; **P < 0.01; ns, not significant.

We next analyzed the phenotypes of core93-specific CD8+ T cells and observed that CT mice showed no significant differences in CD43 expression on total core93-specific CD8+ T cells in the liver and spleen compared to NT and AT mice (Figure 6A). However, the donor core93-specific CD8+ T cells in the liver of CT mice showed significantly lower CD43 expression than those of NT and AT mice (Figure 6B). Similar to the observation in total CD8+ T cells, no significant differences in T_EM_ and T_CM_ differentiation of total, donor, and recipient core93-specific CD8+ T cells were found between CT and NT mice (Figures 6C,D). CT mice showed significantly lower T_EM_ frequencies of total and donor core93-specific CD8+ T cells and significantly lower T_CM_ frequencies of total, donor, and recipient CD8+ T cells in the spleen than AT mice (Figures 6C,D).

Taken together, these results indicate that exhausted HBV-specific CD8+ T cells from CH mice are less activated than naive or memory CD8+ T in the liver after exposure to HBV in an acute activation immune environment. During the course, the differentiation status of exhausted HBV-specific CD8+ T cells is similar to that of naive CD8+ T cells, but different from that of non-exhausted memory CD8+ T cells of AR mice.

### Exhausted HBV-Specific CD8+ T Cells Maintain Functional Unresponsiveness after HBV Reexposure in an Acute Activation Immune Environment

**Figure 7:**
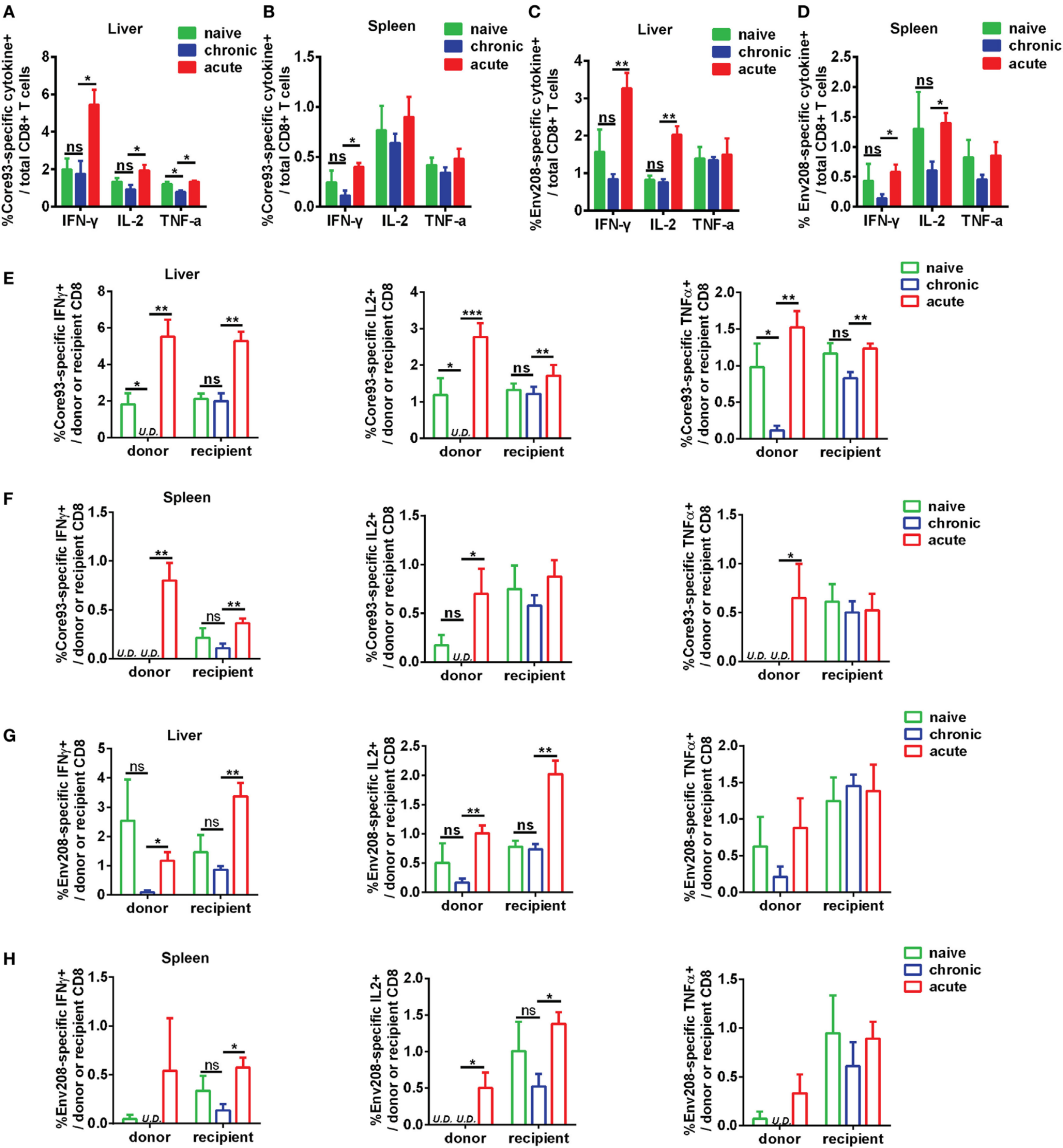
Analysis of effector function of hepatitis B virus (HBV)-specific CD8+ T cells after cell transfer and HBV hydrodynamic injection (HI). Intrahepatic lymphocytes and splenocytes were separated from NT, CT, and AT mice at 21 days post HI. Cells were stimulated by HBV core93-100 peptide or env208-216 peptide for 5 h and were stained for intracellular cytokines interferon (IFN)-γ, IL2, and tumor necrosis factor (TNF)-α. The frequencies of IFN-γ, IL2, and TNF-α producing total **(A–D)**, donor, and recipient **(E–H)** CD8+ T cells in response to HBV core or envelope antigen stimulation in the liver and the spleen were measured by flow cytometry. Data are representative of three independent experiments. Statistics analysis was performed by Kruskal–Wallis test followed by Dunn’s *post hoc* tests. U.D., undetectable. *P < 0.05; **P < 0.01; ***P < 0.001; ns, not significant.

To further characterize the antiviral responses of engrafted CD8+ T cells, we examined the production of effector cytokines IFN-γ, IL-2, and TNF-α by donor and recipient CD8+ T cells in response to HBV core93 and env208 epitope peptides stimulation. The differences in general HBV core and envelope antigen-specific CD8+ T cell responses between CT and NT mice in the liver and the spleen were not significant, except that the total CD8+ T cells of CT mice produced less TNF-α than did the cells of NT mice in response to core93 peptide stimulation in the liver (Figures 7A–D). The total CD8+ T cells of AT mice produced significantly increased IFN-γ (core93: liver and spleen, env208: liver and spleen), IL-2 (core93: liver, env208: liver and spleen), and TNF-α (core93: liver) than those of CT mice (Figures 7A–D). Similar differences in IFN-γ, IL-2, and TNF-α production by the recipient CD8+ T cells of NT, CT, and AT mice were observed (Figures 7E–H). In contrast, the donor CD8+ T cells of CT mice in the liver showed complete absence of IFN-γ and IL-2 production and significantly decreased TNF-α production in response to core93 peptide stimulation compared to those of NT mice (Figure 7E, Figure S5A in Supplementary Material). Compared to the donor CD8+ T cells of AT mice, the cells of CT mice produced significantly decreased IFN-γ (core93: liver and spleen, env208: liver), IL-2 (core93: liver and spleen, env208: liver and spleen), and TNF-α (core93: liver and spleen) (Figures 7E–H; Figure S5B in Supplementary Material).

These results indicate that functionally exhausted HBV core93-specific CD8+ T cells from chronic infection maintain functional unresponsiveness in the liver after HBV reexposure in an acute activation immune environment.

## Discussion

Chronic virus infections start with an acute phase during which virus-specific T cells massively expand and acquire effector functions (38, 39). Failure to eliminate the virus results in a prolonged infection phase during which T cells are continuously exposed to antigen and gradually lose effector functions, such as secretion of antiviral cytokines (40, 41). These functionally exhausted T cells express high levels of inhibitory molecules such as PD-1 and CTLA-4, which are thought to contribute to the T cell dysfunction and chronicity of the infection (42, 43). Blockade of the function of these inhibitory molecules was initially seen as a way of restoring the antiviral function of exhausted T cells (44). However, recent more detailed investigations, mostly in LCMV infection models, indicated that T cell exhaustion represents a stable differentiation state and blocking inhibitory pathways has only temporary and minimal effect on remodeling the state of the cells (45, 46). The retention of the exhausted phenotype was even seen when T cells from chronically LCMV-infected mice were transferred into naive mice and expanded in an acute infection (47). Consistently, we found in the current study that transferring functionally exhausted HBV-specific CD8+ T cells into naive mice and exposing them to an immune activation environment of acute infection failed to restore the antiviral effector function of these cells. This finding indicates that so-called “checkpoint” blockade alone presumably does not lead to a restoration of the potent type of immune response seen in acute infection. In fact, it has been demonstrated by us previously that during chronic hepadnaviral infection, PD-L1 blockade alone showed no effect on restoring viral specific T cell response and controlling viral infection (33). Strategies of blockade of multiple inhibitory effectors, such as combining regulatory T cell depletion with PD-1 and Tim-3 blockade, may be required for the effective restoration of antiviral T cell response in the chronic phase of viral infection as we have shown in Friend virus-infected mice (48).

The association of HBV persistence with a classic phenotype of CD8 T cell exhaustion was not observed in our current study. However, only the expression of inhibitory receptors PD-1, CTLA-4, and LAG-3 was examined, while it remains unclear whether other T cell exhaustion markers such as Tim-3 and 2B4 are upregulated in these T cells. Interestingly, recent studies in mouse noroviruses (MNV) infection have also demonstrated that virus-specific CD8+ T cells from chronic MNV infection showed a decrease of PD-1 expression compared to T cells from mice that resolved MNV infection (49, 50). These MNV-specific tissue-resident T cells retained the ability to expand and were highly functional, yet, they appeared ignorant of ongoing viral replication (50). Further studies are needed to fully characterize the connection between T cell exhaustion phenotypes and their antiviral functions during chronic virus infection.

The results of studies in LCMV infection suggest that exhausted virus-specific CD8+ T cells can still contain viral replication during chronic viral infection (12, 13). After transfer into naive mice, these cells robustly proliferated and controlled viral infection (47). Different to these observations in LCMV infection models, we found that functionally exhausted HBV-specific CD8+ T cells showed no contribution to the control of HBV replication *in vivo* even in an acute-resolving infection setting. Thus, our result suggested that the functional exhaustion status of CD8+ T cells may vary during different chronic virus infections and restoring effector function of HBV-specific CD8+ T cells during chronic HBV infection may request treatments more than removal of immune suppression environment.

Previous studies showed that most T cells transferred from mice with chronic infection into naive host are lost within 2–3 weeks (51, 52). In the current study, a significant number of donor cells were found in the liver and spleen at 7 and 28 days after transfer, indicating the reexpansion of these cells in response to acute HBV infection. This is further supported by the observation that the engrafted CD8+ T cells from CH mice upregulated Ki67 expression in response to HBV challenge. Thus, we conclude that chronic HBV infection generated exhausted CD8+ T cells with population-reexpansion potential, although it was profoundly impaired compared with that of memory T cells of resolved HBV replication, which suggested that not all virus-specific CD8+ T cells undergo terminal differentiation during chronic HBV infection. It has been reported that two distinct states of virus-specific CD8+ T cells, which differentially express the T-box transcription factors T-bet and Eomesodermin (Eomes), exist in chronically infected mice and humans (53, 54). In response to antigenic stimulation, T-bet^hi^ cells exhibit higher proliferative rates and give rise to Eomes^hi^ cells, which are more exhausted (55). Thus, the reexpansion of HBV-specific CD8+ T cells from CH mice after transfer may be due to the terminal differentiation of progenitor T-bet^hi^ cells to progeny Eomes^hi^ cells. Analysis of T-bet and Eomes expression dynamics in exhausted T cells during chronic HBV infection is needed for future studies.

In conclusion, we have demonstrated here that although functionally exhausted HBV-specific CD8+ T cells of chronic replication retained proliferative ability, it was profoundly impaired compared with that of the non-exhausted memory CD8+ T cells of HBV resolvers. These cells showed less effector differentiation, and maintained less activated phenotypes, an absence of effector cytokine production and poor antiviral function after exposure to acute HBV infection setting. The reconstitution of antiviral function of exhausted HBV-specific CD8+ T cells may request powerful immunological means more than switch immune environment.

## Ethics Statement

All animal experiments were conducted in a BSL-2 laboratory facility in accordance with the Guide for the Care and Use of Laboratory Animals and were reviewed and approved by the Institutional Animal Care and Use Committee at Tongji Medical College, Huazhong University of Science and Technology, China (IACUC Number: 612).

## Author Contributions

DY and JLiu designed the experiments; QW, WP, JLiu, JLuo, DZ, and YLiu performed the experiments; YLu, XF, XY, UD, and ML provided experiment materials; QW, WP, and JLiu analyzed the data; JLiu and QW drafted the manuscript. All authors read and approved the final manuscript.

## Conflict of Interest Statement

The authors declare that the research was conducted in the absence of any commercial or financial relationships that could be construed as a potential conflict of interest.

## Abbreviations

HBV, hepatitis B virus; HCC, hepatocellular carcinoma; AR mice, acute-resolving HBV replicating mice; CH mice, chronically HBV replicating mice; CT mice, mice transferred with CD8+ T cells from CH mice; AT mice, mice transferred with CD8+ T cells from AR mice; NT mice, mice transferred with naive CD8+ T cells; HI, hydrodynamic injection; dpi, days postinjection; LCMV, lymphocytic choriomeningitis virus; Eomes, Eomesodermin; SPF, specific pathogen-free; IHLs, intrahepatic lymphocytes; MACS, magnetic-activated cell sorting; PCR, polymerase chain reaction; IFN, interferon; IL, interleukin; TNF, tumor necrosis factor.
