# Supplementary material for: Hepatitis B Virus-Specific CD8+ T Cells Maintain Functional Exhaustion after Antigen Reexposure in an Acute Activation Immune Environment
Source: Front Immunol. 2018 Feb 12;9:219. doi: 10.3389/fimmu.2018.00219 (PMC5816053; doi:10.3389/fimmu.2018.00219)
Supplement: Supplementary file 1 [file data_sheet_1.docx]

Supplementary Material

HBV-specific CD8+ T cells maintain functional exhaustion after antigen reexposure in an acute activation immune environment

Qin Wang^1,2^*, Wen Pan^1^*, Yanan Liu^1^, Jinzhuo Luo^1^, Dan Zhu^1^, Yinping Lu^2^, Xuemei Feng^2^, Xuecheng Yang^2^, Ulf Dittmer^3^, Mengji Lu^3^, Dongliang Yang^1,2^*, Jia Liu^1,2^*

^1^*Department of Infectious Diseases, Union Hospital, Tongji Medical College, Huazhong University of Science and Technology, Wuhan 430022, China*

^2^*Institute of Infection and Immunology, Union Hospital, Tongji Medical College, Huazhong University of Science and Technology, Wuhan 430022, China*

^3^*Institute for Virology, University Hospital of Essen, University of Duisburg-Essen, Essen 45147, Germany*

*These authors have contributed equally to this article.

**Correspondence to:**

Prof. Jia Liu, email: [jialiu77@hotmail.com](mailto:jialiu77@hotmail.com)

Prof. Dongliang Yang, email: [dlyang@hust.edu.cn](mailto:dlyang@hust.edu.cn)


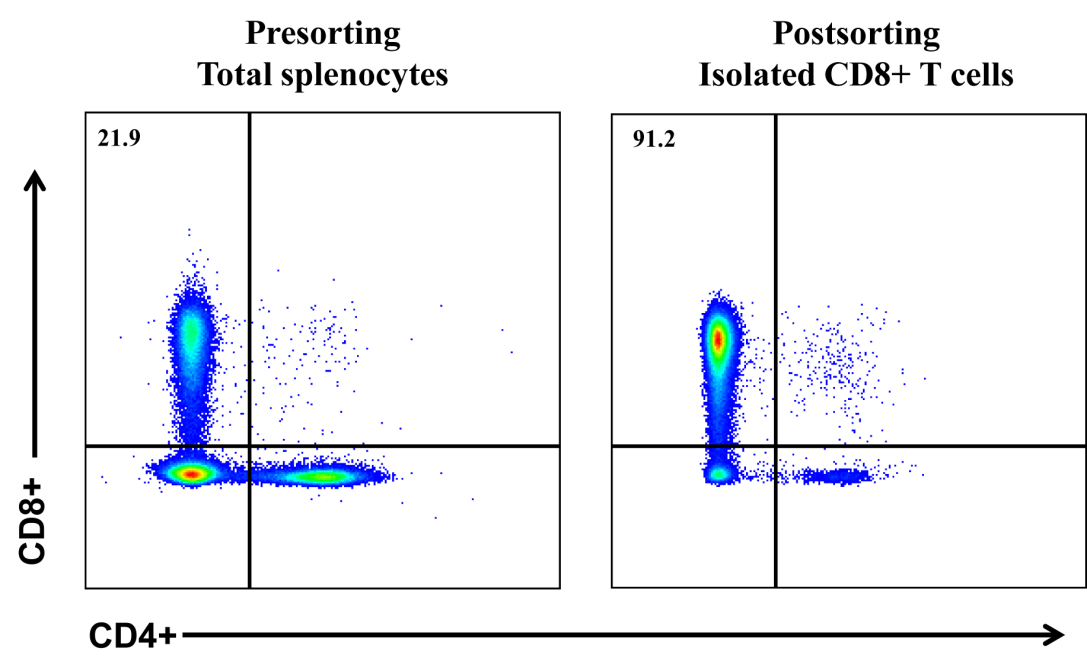


**Figure S1. The purity of CD8+ T cells after isolation.** The frequencies of CD8+ T cells before and after purification were analyzed by flow cytometry.





**Figure S2. Determination of the background levels of dimer staining and ICC assay.** HBV core93-specific dimer staining (A) and ICC assay for IFNγ, IL-2 and TNFα (B) were performed in splenocytes from naive C57BL/6 mice.

**
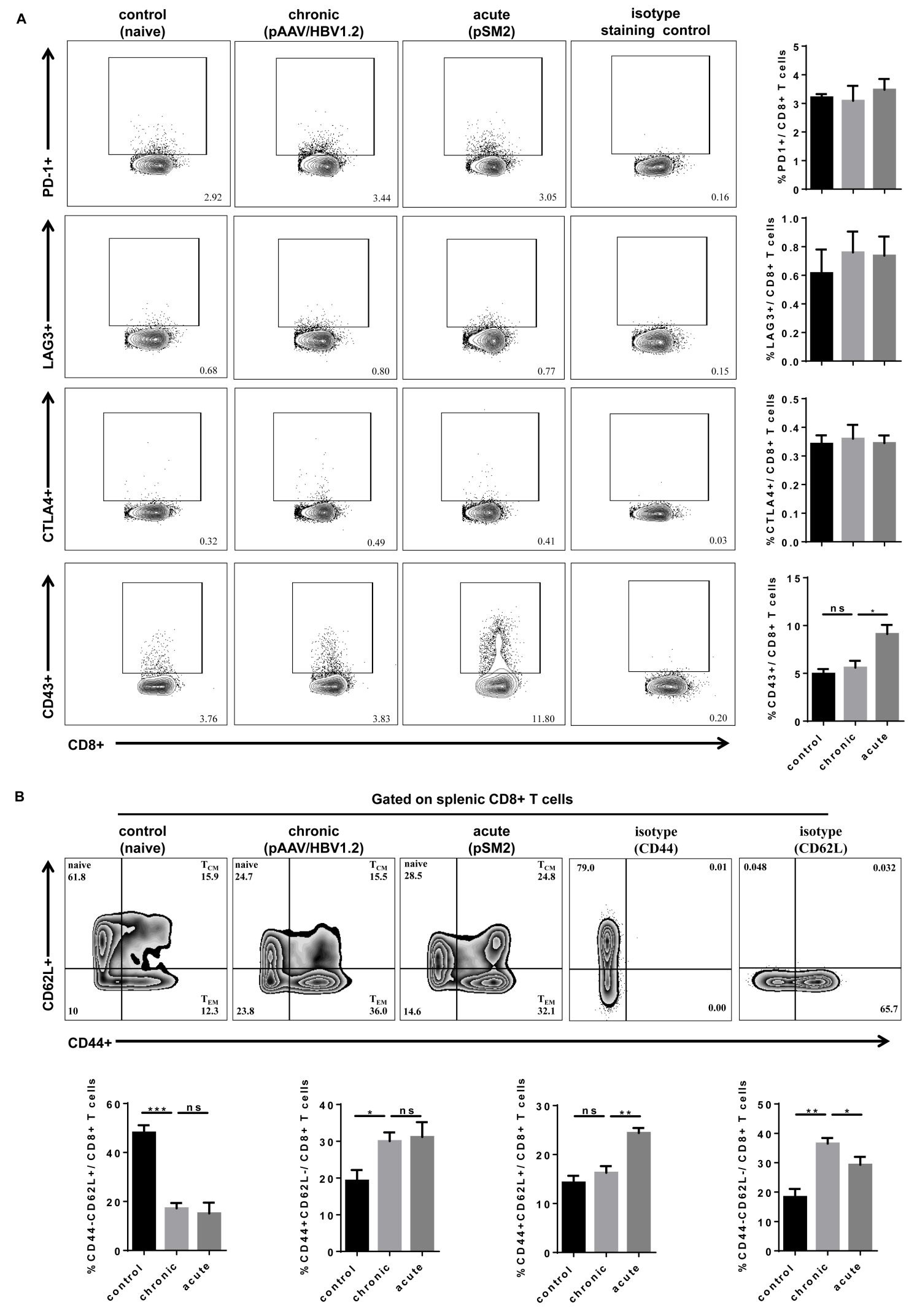
**

**Figure S3. Comparison of CD8+ T cells phenotype between chronically and acute-resolving HBV replicating mice.** C57BL/6 mice were hydrodynamically injected with either pAAV/HBV1.2 or pSM2 plasmid. Splenic CD8+ T cells were analyzed for the expression of activation / exhaustion markers at 21 days post HI. Percentages of (A) PD-1+，LAG-3+, CTLA-4+, CD43+, (B) CD44-CD62L+ (naive), CD44+CD62L-(T_EM_), CD44+CD62L+ (T_CM_) and CD44-CD62L- CD8+ T cells were detected by flow cytometry. Data are representative of three independent experiments. One-way ANOVA followed by LSD test were applied. *, *P*<0.05; **, *P*<0.01; ***, *P*<0.001; ns, not significant.


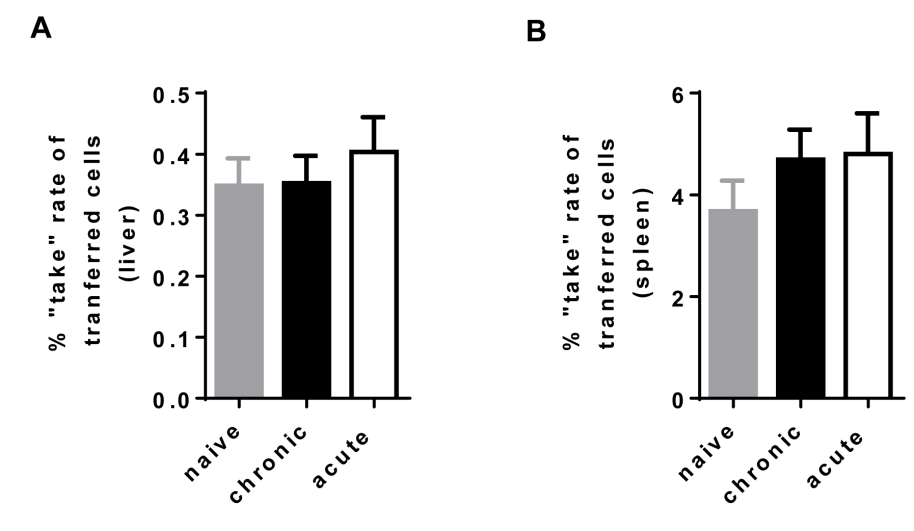


**Figure S4: Determination of the “take” rates of engrafted cells.**  5×10^6^ splenic CD8+ T cells were transferred into naive host mice. The absolute numbers of engrafted CD8+ T cells in the liver and the spleen were counted 1 day after cell transfer. The “take” rates of engrafted cells in the liver (A) and the spleen (B) were calculated using following formula: percentage of cell take = numbers of CD45.2+ cells in the organ / total number of transferred cells × 100.


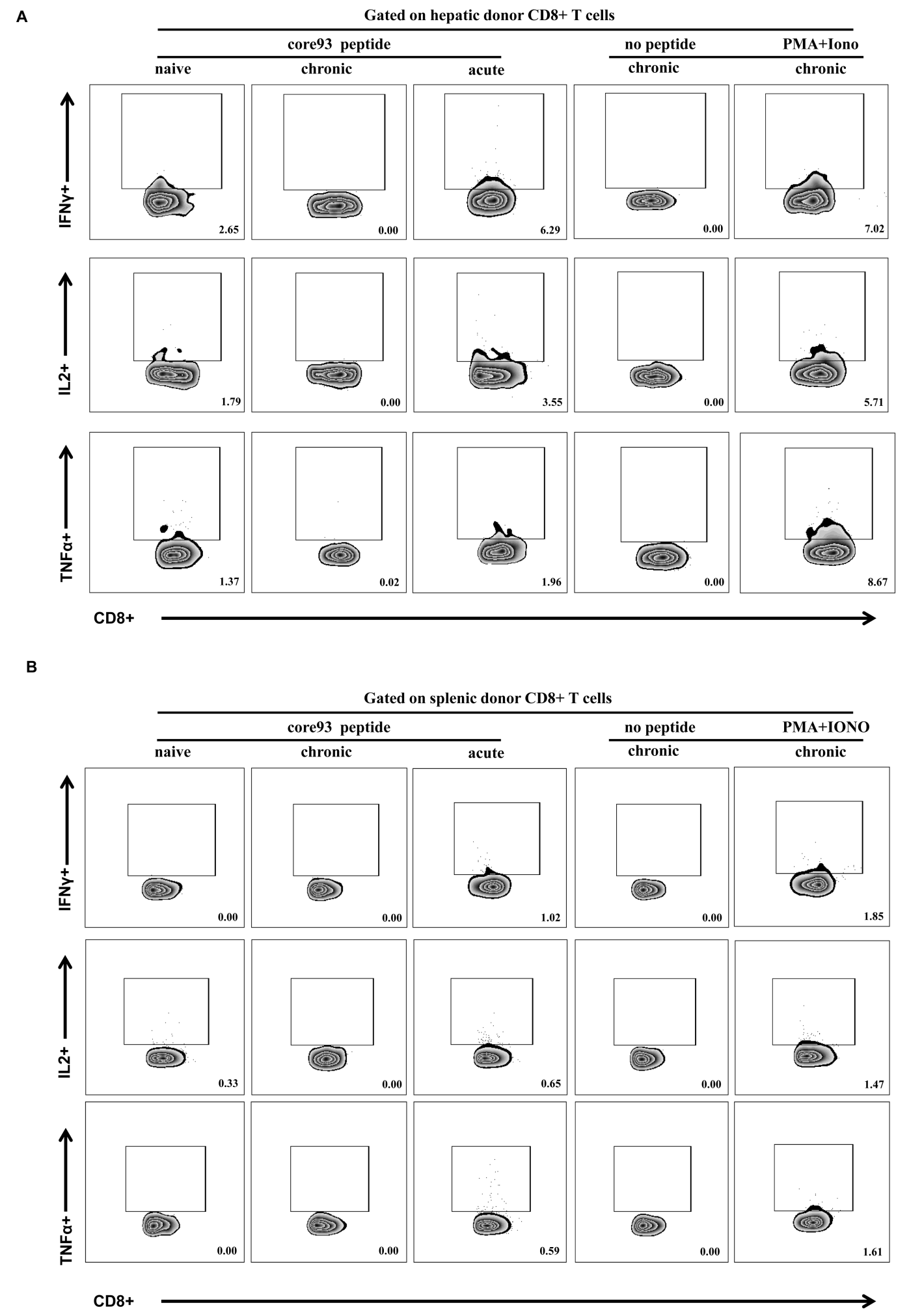


**Figure S5. Analysis of effector function of HBV-specific CD8+ T cells after cell transfer and HBV hydrodynamic injection.** Intrahepatic lymphocytes and splenocytes were separated from NT, CT and AT mice at 21 days post HI. Cells were stimulated by HBV core93-100 peptide or env208-216 peptide for 5h and were stained for intracellular cytokines IFN-γ, IL2 and TNF-α. The representative dot plots of IFN-γ, IL2 and TNF-α producing donor-derived CD8+ T cells in the liver (A) and the spleen (B) were shown.
